# RERAS—robotic colorectal resections and ERAS® in older adults: optimizing recovery or adding complexity?

**DOI:** 10.3389/fsurg.2025.1638414

**Published:** 2025-12-03

**Authors:** M. El-Ahmar, J. Hardt, C. Reissfelder, J.-P. Ritz, F. Peters, S. Seyfried

**Affiliations:** 1 Department of Surgery, Universitätsmedizin Mannheim, Medical Faculty Mannheim, Heidelberg University, Mannheim, Germany; 2 Department of General and Visceral Surgery, Helios Hospital Schwerin, University Campus Medical School Hamburg, Schwerin, Germany

**Keywords:** robotic surgery, ERAS®, colorectal resections, older adults, perioperative management

## Abstract

**Purpose:**

Robot-assisted surgery (RAS) has established itself as a minimally invasive approach in colorectal surgery, although evidence on its integration with Enhanced Recovery After Surgery (ERAS®) protocols in older patients remains limited. This study aims to describe short-term outcomes of RAS combined with a perioperative treatment according to the ERAS® protocols in patients ≥70 years.

**Methods:**

This retrospective analysis of a prospectively maintained database includes all patients aged ≥70 years who underwent robotic colorectal resections at two German colorectal cancer centers between January 2019 and April 2024, managed perioperatively according to the ERAS® protocols. Primary endpoints were the patients' short-term perioperative outcomes, including duration of surgery, conversion rate, postoperative Intermediate-Care-Unit (IMC) admission, postoperative complications according to Clavien-Dindo, anastomotic leak and reoperation rate, length of hospital stay, and compliance to ERAS® guidelines. Results are presented descriptively without a comparator arm.

**Results:**

A total of 161 patients (99 colon resections and 62 rectal resections) were included over the study period. Median duration of surgery was 153 (IQR: 130–197) minutes for colon and 243 (IQR: 120–467) minutes for rectal resections. Conversion rates were 1% and 4.8% respectively. Postoperative IMC admission was required in 9.1% (9 Patients) after colon and 12.9% (8 Patients) after rectal-resections, based on individual clinical assesement. Anastomotic leaks occurred in 7 cases (7%) following colon resections, with a total reoperation rate of 10%. Among rectal resections, the anastomotic leakage rate was 9.7% (6 cases) with a total reoperation rate of 16.1%. ERAS® compliance was 91.3% for colon- and 85% for rectal resections. Within the rectal cohort, postoperative complications were associated with a substantially lower perioperative ERAS® compliance compared to patients without complications (73.3% vs. 90.7%). Hospital stay was 5 days (IQR: 4–6 days) for colon- and 6 days (IQR: 5–11 days) for rectal resections. The 30-day readmission rate was 4% (4 cases) for colon and 8% (5 cases) for rectal resections.

**Conclusion:**

The integration of RAS colorectal surgery within ERAS® protocols appears feasible and is associated with acceptable short-term outcomes in elderly and comorbid patients. Nonetheless, these results should be interpreted as descriptive observations rather than inferential evidence.

## Introduction

As in all medical fields, surgery is continuously evolving. New treatment methods and surgical techniques are regularly introduced to optimize patient care. Colorectal surgery is also experiencing such a transformation, shifting from traditional open procedures to minimally invasive techniques.

The advantages of minimally invasive surgery are clear: patients experience significantly less surgical trauma and faster postoperative recovery (1). In recent years, the purely laparoscopic technique has been further advanced with the increasing accessibility of robot-assisted surgery (RAS), elevating the approach to a new level. Three-dimensional visualization of the surgical field with tenfold magnification for fine-structure recognition, combined with the 360° maneuverability of micro-instruments equipped with tremor filters, provides surgeons with enhanced tools to reduce patient trauma and accelerate recovery.

Equally important to advancing surgical methods and related technology is the optimization of perioperative management to accelerate postoperative recovery. The ERAS® (Enhanced Recovery After Surgery) protocols describe a multimodal and interdisciplinary treatment approach that utilizes evidence-based measures to optimize patient recovery after surgery (2). The ongoing relevance of this topic, more than 25 years after its initial publication by Danish surgeon Henrik Kehlet (3), is underscored by the release of the new German S3 guideline for perioperative management in gastrointestinal tumors (POMGAT) in November 2023 (4) and the steadily increasing number of certified ERAS® centers in Germany.

This is if utmost importance, especially because of the aging population with an increasing number of older patients presenting for major surgery. The prevalence of frailty and age-related physiological changes significantly influence the postoeprative outcomes. Therefore, in order to minimize the adverse postoperative outcomes in frail patients during hospitalization, it is critical to lower the stress response (5).

Minimally invasive surgery remains a core component of the ERAS® protocol and it has shown positive outcomes for elderly patients (6, 7). However, the ERAS® Society's guidelines focus primarily on the laparoscopic approach, as its compatibility with ERAS® has been thoroughly demonstrated. Currently, there are no specific recommendations for the application of RAS within the ERAS® framework, and data on the feasibility of combining RAS with a multimodal perioperative ERAS® protocol in elderly patients is limited. Initial studies indicate that the ERAS® protocol can significantly reduce stress for this particular patient population (8, 9). However, before a solid recommendation can be made in favor of any specific surgical technique, further analyses are necessary. It must first be determined whether the robotic approach—despite the substantial learning curve for the entire surgical team—achieves the anticipated positive effect in combination with the ERAS® protocol for this patient demographic.

The aim of this study is therefore to evaluate the synergistic effect of minimally invasive robotic surgery and the multimodal Enhanced Recovery protocol, particularly in older patients who tend to be more vulnerable to the perioperative stress response, in a dual-center analysis.

## Material and methods

All patients aged ≥70 years undergoing elective colorectal resection between January 2019 and April 2024 were included in this retrospective analysis of a prospectively maintained database. Procedures were performed using a Da Vinci® Xi robotic system and perioperative management followed institutional ERAS® protocols. The robotic approach was applied whenever platform capacity allowed. The age cut-off of 70 years was chosen because it lies slightly above the statutory retirement age in Germany (67 years) and therefore represents a group generally regarded as elderly in the national healthcare setting.

Exclusion criteria were patients younger than 70 years, emergency colorectal resections, and procedures not performed robotically or not managed according the ERAS® protocols. All operations were conducted by certified colorectal surgeons at accredited colorectal cancer centers. In cases of malignancy, oncological resections were performed in accordance with institutional standards. For colon cancer, a complete mesocolic excision (CME) was routinely performed to ensure en-bloc resection of the mesocolon with intact fascial planes. For rectal cancer, a total mesorectal excision (TME) was performed in both anterior and abdominoperineal resections to achieve optimal oncological outcomes.

The Helios Kliniken Schwerin and the University Medical Center Mannheim were the first two certified ERAS® centers of excellence in Germany. Certification by the ERAS® Society is associated with multiple perioperative parameters subject to regular quality control. Before implementing the ERAS® program, extensive training was provided to all key personnel, including surgeons, anesthesiologists, nurses, and physiotherapists. This was intended to ensure that patient recovery was structured, transparent, and as straightforward as possible for both patients and staff, allowing the treatment pathway to function independently of specific personnel assignments.

To prepare patients optimally for their hospital stay and ERAS®-based treatment, each patient received preoperative counseling from a physician within the in-house ERAS® team and an ERAS® nurse. This comprehensive preparation included explanations of the ERAS® protocol and a detailed overview of the hospital stay, covering topics such as prehabilitation, the minimally invasive robotic approach using the Da Vinci® Xi system, early oral intake, early mobilization, and personalized analgesic therapy.

All patients received a standardized preoperative bowel preparation consisting of a polythelyne glycol-based solution, followed by oral antibiotic prophylaxis with paromomycin (1 g). In patients presenting with stenosing tumors causing a consecutive ileus, mechanical bowel preparation was omitted (11 patients undergoing colon resection and 18 patients undergoing rectal resection). In patients who had received neoadjuvant oncological treatment, no additional chemoprohylaxis was administered.

To prevent information overload and to systematically document and scientifically support the hospital stay, both clinics independently developed a patient diary. Documentation was maintained by an ERAS® nurse, who entered data into the ERAS®-specific documentation system, the ERAS® Interactive Audit System (EIAS). All data necessary for this publication were sourced from the EIAS.

For the perioperative data analysis, baseline demographic and clinical variables were extracted from the EIAS and included age, gender, and preoperative risk classification according to the American Society of Anesthesiologists (ASA). Major comorbidities documented in the database were Diabetes Mellitus, severe heart disease (e.g., severe coronary artery disease, heart failure ≥ NYHA III) and severe pulmonary disease (e.g., COPD GOLD ≥ III). Frailty indices and Composite Comorbidity Scores were not available within the EIAS and therefore could not be assessed. Patient diagnoses were limited to benign and malignant entities. A distinction was also made between colon and rectal resections, with the extent of resection documented as per EIAS standards.

The colon resections included the following procedures:

- Ileocecal resection/right hemicolectomy
- Sigmoid resection
- Left hemicolectomy
- Reversal of Hartmann's situation
- Other types of colon resections

The rectal resections included the following procedures:

- (Low) Anterior resection of the rectum
- Abdominoperineal resections

To assess short-term perioperative outcomes, the following target parameters were collected:

- Duration of surgery
- Conversion rate
- Intraoperative blood loss
- IMC stay in days
- Postoperative complications according to Clavien-Dindo
- Anastomotic leakage rate
- Reoperation rate
- Compliance to ERAS® guidelines
- Length of hospital stay in days
- Readmission rate within the first 30 days
- Complications were classified according to the Clavien-Dindo score (10). Minor complications included Grades I to IIIa, while major complications included Grades IIIb to V according to the Clavien-Dindo classification. The reoperation rate was limited to two specific causes in EIAS: anastomotic leak and other causes.

ERAS® compliance was defined as the proportion of applicable ERAS® items fulfilled across the perioperative period, as documented in the EIAS by trained ERAS® nurses. Items not applicable were excluded from the denominator.

Continous variables were presented as median and Interquartile Range (IQR) and categorical variables as absolute numbers and percentages. Given the descriptive design of this dual-center analysis and the absence of a comparator arm, no univariable or multivariable analysis were performed. The study was not powered to identify independent predictors of postoperative outcomes. Therefore, all results ar reported descriptively.

## Results

The general patient characteristics are summarized in Table 1.

**Table 1:** Patient characteristics.

Cohort characteristics	Robotic colon resections *n* = 99	Robotic rectal resections *n* = 62
Age (years)	77 (70–93)	77 (70–89)
Gender (Male:Female)	48:51	18:44
BMI (kg/m^2^)	26.80 IQR: 24.06–29.49	25.82 IQR: 23.58–29.04
ASA-Score		
1	2 (2%)	1 (1.6%)
2	32 (32.3%)	20 (32.3%)
3	65 (65.7%)	38 (61.3%)
4		3 (4.8%)
Co-morbidities		
Diabetes Mellitus	25 (25.3%)	10 (16.1%)
Severe heart disease	7 (7.1%)	3 (4.8%)
Severe Pulmonary disease	2 (2%)	–
Preoperative WHO performance status		
Asymptomatic	89 (89.9%)	52 (83.9%)
Symptomatic—ambulant	9 (9.1%)	9 (14.5%)
Symptomatic <50% in bed (in bed during the day)	1 (1%)	1 (1.6%)
Diagnosis (Benign:Malignant)	29:70	5:57
Neoadjuvant oncolocigal treatment	2 (2%)	34 (54.8%)

BMI, Body-mass-index; ASA-score, amercican society of anesthesiologists risk classification score; WHO, World-health-organization.

### Specific results for colon resections

Of the 99 colon resections, 46 cases (46.5%) were right-sided (including ileocecal resection or right hemicolectomy), while 41 cases (41.4%) involved left-sided resections (28 sigmoid resections and 13 left hemicolectomies). Additionally, 2 cases (2%) involved restoration of continuity in Hartmann's situation, and 10 cases (10.1%) involved other types of colon resections. Among colon resections, oncological procedures involving CME were performed in 70 patients.

The duration of surgery for Ileocecal resection/right hemicolectomy was 161 min [interquartile range (IQR) 132–196 min], with no conversion and an intraoperative blood loss of 50 mL (IQR: 50–150 mL).

For sigmoid resections the duration of surgery was 174 min (IQR 152–187 min), with a conversion rate of 3,6% (1 case) and an intraoperative blood loss of 125 mL (IQR: 50–237 mL).

For left hemicolectomy the duration of surgery was 191 min (IQR 160–203 min), with no conversion and an intraoperative blood loss of 60 mL (IQR: 25–100 mL).

To achieve a measurable number of cases, reversal of Hartmann's situation and other colon resections were combined. The duration of surgery for these cases was 136 min (IQR 119–166 min), with no conversions and an intraoperative blood loss of 62 mL (IQR: 49–200 mL).

Six patients following a reoperation due to an anastomotic leakage and two patients due to a reoperation because of an incarcerated trocar hernia and intraabdominal hemorrhage were transferred to the IMC postoperatively. No patient was transferred to the IMC after the initial operation.

During the postoperative period, 10 patients (10.1%) experienced major and 7 patients (7.1%) minor complications. Minor complications included 2 cases of postoperative paralysis, 2 cases of postoperative wound infections and 3 cases of postoperative nausea and vomiting. All minor complications were treated conservatively. Anastomotic leakage occurred in 7 cases (7%), all of which required reoperation. In an additional 2 cases (2%) an explorative laparoscopy was performed, thus ruling out an intraabdominal pathology. 1 patient required reoperation due to an incarcerated trocar site hernia bringing the total reoperation rate to 10%. 2 Surgical Site Infections (SSI) were detected within 30 days postoperatively, including during follow-up, both of which were managed with local therapy.

When ERAS® compliance was evaluated in relation to postoperative morbidity, the median perioperative compliance rate was 90.9% in patients with minor and 88.9% in those with major-complications.

The intravenous fluid administration on the day of surgery was 2,300 mL (IQR: 1,800–3,000 mL). Time to first postoperative bowel movement was 2 days (IQR: 1–3 days), while the time until patients tolerated oral intake of solid food was 1 day (IQR: 1–2 days). Pain was managed with oral analgesics by a median of 1 day postoperatively (IQR: 1–2 days). Early mobilization was achieved on the first postoperative day (median 4 h, IQR: 4–5), increasing to 6 h on the second (IQR: 5–6 h) and third postoperative days (IQR: 5–7 h).

Overall adherence to ERAS® guidelines for colon resections was 91.3% (IQR: 86.4%–95,6%). The median hospital stay was 5 days (IQR: 4–6 days), and 4 patients (4%) required readmission within 30 days postoperatively. 2 Patients died with within 30 days postoperatively (2%).

### Specific results for rectal resections

Of the 62 rectal resections, 52 patients (83.9%) had anterior resection of the rectum and 10 (16.1%) an abdominoperineal resection. In the rectal cohort, TME was performed in 57 patients. A diverting loop ileostomy was conducted in 25 patients to protect the distal anastomosis.

The duration of surgery for (low) anterior rectal resections was 234 min (IQR: 202–292 min) with a conversion rate of 3,8% (2 cases) and an intraoperative blood loss of 175 mL (IQR: 100–400 mL).

The duration of surgery for abdominoperineal resections was 245 min (IQR: 220–315 min) with a conversion rate of 10% (1 case) and an intraoperative blood loss of 400 mL (IQR: 175–650 mL).

Five patients (8%) following a reoperation due to an anastomotic leakage were transferred to the IMC postoperatively. Three patients (3%) were transferred to the IMC following the initial operation due to other causes like blood loss up to 900 mL or the need for intravenous cathecolamines.

Postoperatively, complications were seen in a total of 26 patients (42%). Minor complications included. 4 Patients with postoperative paralysis, 4 Patients with urinary retention/renal failure, 2 Patients with a urinary tract infection and 2 Patients with a postoperative hematoma and Pneumonia. 4 Patients received an Endoluminal Vacuum assisted closure (EndoVac) treatment due to an anastomotic leakage.

In 2 patients the anastomosis was taken down and an end-stoma was constructed. An additional 8 cases (12.9%) required reoperation for other reasons, resulting in an overall reoperation rate of 16.1%.

When ERAS® compliance was evaluated in relation to postoperative morbidity, the median perioperative compliance rate was 90.7% in patients with minor and 73.3% in those with major-complications.

The intravenous fluid administration on the day of surgery was 3,150 mL (IQR: 2,300–4,180 mL). The time to first postoperative bowel movement was 1 day (IQR: 1–2 days), while the time to tolerate solid food intake was also 1 day (IQR: 1–2 days). Pain was managed with oral analgesics by a median of 2 days postoperatively (IQR: 1–3 days). Early mobilization was achieved with a median of 4 h on the first postoperative day (IQR: 2–4 h), increasing to 5 h on the second (IQR: 3–6 h) and 6 h on the third postoperative day (IQR: 3–6 h).

Overall, ERAS® adherence for rectal resections was 85,7% (IQR: 73,91%–95,24%). The median hospital stay was 6 days (IQR: 5–11 days), and 5 patients (8%) required readmission within the first 30 days postoperatively. The overall mortality rate within 30 days postoperatively was 3.2% (2 cases).

The specific outcomes of the study are summarized in Table 2.

**Table 2:** Perioperative outcomes.

Intra- and postoperative variables	Robotic colon resections *n* = 99	Robotic rectal resections *n* = 62
Duration of surgery (in minutes)	153 (130–197)	243 (120–467)
Conversion rate	1 (1%)	3 (4,8%)
Intraoperative blood loss (in mL)	78 (50–200)	175 (100–400)
Postoperative IMC admission	9 (9.1%)	8 (12.9%)
(length of stay in days)	4 (2–8)	7 (2–14)
Postoperative complications (Clavien-Dindo)		
IIIb–V (Major)	10 (10.1%)	10 (16%)
I–IIIa (Minor)	7 (7.1%)	16 (26%)
Anastomotic leakage rate	7 (7%)	6 (9.7%)
Surgical Site Infection	2 (2%)	–
Reoperation rate	10 (10%)	10 (16.1%)
ERAS®-adherence rate	91.3% (86.4%–95.6%)	85.7% (73.91%–95.24%)
Hospital length of stay (in days)	5 (4–6)	6 (5–11)
Readmission rate (Within 30 d postop.)		
Yes	4 (4%)	5 (8%)
No	95 (96%)	57 (92%)
Mortality rate	2 (2%)	2 (3.2%)

IMC-stay, Intermediate Care Unit length of stay; ERAS®, Enhanced recovery after surgery.

## Discussion

Particularly for older patients, minimizing the stress response to surgery is essential. The ERAS® protocol encompasses over 30 individual steps that collectively target rapid patient recovery (8). A core component of ERAS® is minimally invasive surgery, for which the ERAS Society provides a clear recommendation for colorectal resections in the guidelines published in 2018 (10). However, this recommendation currently only applies to conventional laparoscopy. Despite early studies showing positive results of robotic colorectal surgery (11, 12), robotic surgery has yet to be incorporated into the ERAS® Society guidelines or the German S3 guidelines for colorectal cancer (10, 13). This is largely due to a lack of large-scale studies examining the combination of RAS and multimodal perioperative treatment.

The advantages of robotic surgery which additionally tend to reduce surgical trauma by enhanced vision and precise tissue dissection, should theoretically have a synergetic positive impact on patients treated following the ERAS® protocols. Early studies with a case series of less than 100 patients in total already showed promising synergetic effects of RAS and ERAS® in a mixed patient population (12), which in turn raised the question, if older patients in particular would benefit by the combination of these programs.

Despite the median age of 77 years of our patient population with about two-thirds classified with a severe systemic disease (ASA 3), our analysis proved that the combination of RAS and ERAS® (RERAS) has a positive effect on patients particularly of older age. These findings are significant, especially when we consider the facts that older an multimorbid patients tend to have a higher postoperative non-surgical complication rate than those of younger age. Therefore, an exclusion of older multimorbid patients, particularly of older age, for RAS is not justified as we could demonstrate in our analysis.

By an overall high adherence to the ERAS® protocols, minor postoperative non-surgical complications accounted for only around 7% in colon and less than 20% in rectal resections. By adequately minimizing the pain, early mobilization beginning already in the recovery room, could be achieved for several hours per day. Hence, early toleration of solid food followed by first postoperative bowel movement, led to an enhaced recovery of these patients.

It is known that the duration of surgery is an independent risk factor for patients' short term outcomes after colorectal resections (14). This represents an essential parameter for the older and frail patient population in our study. However, in order to perform save robotic colorectal surgery in an acceptable duration of surgery must be trained (15, 16). A 2023 multicenter study from China reported a median operative time of around 200 min with no conversions in robotic right hemicolectomies (17). In 2023, we could publish the first study on robotic colorectal resections combined with the ERAS® protocol with a larger patient cohort. After continuous training, median operating times for colon resections were 167 min, with a conversion rate of 3.1%; for rectal resections, the median time was 246 min, with a conversion rate of 14.3% (12). In our analysis, despite implementing two fundamentally new treatment methods in two distinct high-volume centers with different colorectal surgeons, operating times and conversion rates were consistent with international benchmarks: 153 min with a conversion rate of 1% for colon resections, and 243 min with a 4.8% conversion rate for rectal resections.

To add, as anesthesiological care has advanced significantly, particularly by using total intravenous anesthesia as used for all patients in our cohort, the benefit of reduced operative time seen in conventional open surgery, as shown by Fujii in 2014 (18), appears to have a less influence on patients' recovery during their hospital stay.

A Study by Tamagawa in 2021 (18) highlighted that increased intraoperative blood loss is associated with higher perioperative morbidity, although this association was not supported by Saleh in 2016 (19). In our study, intraoperative blood loss was approximately 80 mL for colon resections and 175 mL for rectal resections, suggesting minimal blood loss and, per Tamagawa's findings, unlikely to impact postoperative morbidity. However, blood loss was higher in anterior rectal and abdominoperineal resections compared with colon resections. This difference is likely attributable to the higher proportion of patients who had received neoadjuvant chemoradiotherapy, which increases tissue fragility and vascularity, as well as the technical demands and limited working space of pelvic dissection during, especially during the learning curve of robotic TME.

Many hospitals still transfer patients to IMC following colorectal resection for various reasons, including safety concerns or the preoperative epidural catheter (EC) placement because of standard practice—regardless of the surgical approach (open or minimally invasive), aiming to support postoperative pain management and postoperative bowel stimulation (20). This practice is reflected in the annual report of the StuDoQ database of the German Society for General and Visceral Surgery, which reports only around 46% of colon resections and 64% of rectal resections in Germany are performed laparoscopically, with EC placement still common in conventional procedures (21, 22). EC placement frequently results in postoperative hypotension requiring catecholamines, necessitating IMC monitoring (23). In contrast to Celik's 2018 findings, where the median IMC stay following laparoscopic colorectal resections was 1 day (24), nearly 90% of patients, despite the older age and the frailty in our study were postoperatively transferred directly to a general surgical ward, with IMC admission required only in selected cases. This underscores the perioperative stability achieved through a structured multimodal enhanced recovery after surgery program combined with minimally invasive robotic surgery. Clinicallly and economically, the low rate of IMC utilization highlights the benfit of the RERAS-concept, demonstrating its efficiency through reduced postoperative monitoring requirements and optimized rescource utilization, all while maintaining a consistently high standard of patient safety.

One of the most critical indicators of postoperative outcomes is the complication rate. SSI represent the most frequent postoperative complication in colorectal surgery with a range from 9% to 41% (25) and are associated with significant patient burden in terms of pain and impaired recovery. Beyond their clinical impact, SSIs contribute to increased healthcare costs, higher morbidity, prolonged hospitalization, elevated readmission rates, and can lead to severe consequences such as sepsis or even mortality (26). In our cohort, the incidence of SSI was limited to 2% (2 cases) following colon resections, with no occurrences reported after rectal procedures. We attribute this low rate to the synergistic effect of RAS and strict adherence to perioperative ERAS® protocols, both of which were consistently implemented with high compliance throughout the study population.

Higher rates of anastomotic leaks correlate with increased mortality and recurrence rates in cancer patients, as well as with reduced disease-free survival (27–30). According to publications by Dulskas and Kryzauskas, anastomotic leak rates after colorectal resections range from 2% to 19% (31, 32) and have limited sufficient treatment options (33). The German Cancer Society mandates quality thresholds for leak rates of ≤6% for colon and ≤15% for rectal resections at certified colorectal cancer centers in Germany (34). The target for reoperation rates is ≤15% for both. These benchmarks, however, do not adjust for patient age or comorbidities. With our combined approach of multimodal perioperative management and robotic-assisted surgery, our anastomotic leak rate was 7% for colon resections, with a reoperation rate of 10%. For rectal resections, our anastomotic leak rate was below 10%, the reoperation rate was about 15%, likely reflecting the steep learning curve for rectal procedures. Reoperation causes including postoperative bleeding, mechanical obstructions, bowel perforations, and thermal damage, all likely could be avoided in the near future by robotic operating technique refinement.

We achieved ERAS® adherence rates of approximately 90% for colon and 85% for rectal resections. Several studies, including Ni's 2019 meta-analysis of nearly 1,300 patients, and Wei and Catarci's studies in 2020 and 2021, respectively, have confirmed that higher ERAS® adherence correlates with lower postoperative morbidity and shorter hospital stays (35–37). We observed similar trends in our study, with median hospital stays of 5 days for colon and 6 days for rectal resections. Notably, within the rectal cohort, patients who developed postoperative complications demonstrated lower perioperative ERAS® compliance compared to those with minor or no complications (median 73.3% vs. 90.7%). Although this analysis was descriptive and not based on formal statistical testing, the difference appears clinically meaningful and highlights the relationship between protocol adherence and outcome. While the underlying causality cannot be determined from our data, the observation suggests an interdependence between adherence and postoperative course. Complications may hinder full compliance to ERAS® elements, while reduced compliance in turn may reflect the physiological or clinical challenges associated with more complex cases.

The integration of robotic surgery into perioperative multimodal enhanced recovery programs like the ERAS® protocols for elderly patients should be viewed within the broader context of modern geriatric surgical care. While our data confirm that the combination of RAS and ERAS® (RERAS) is feasible in an older population, the abscende of specific recommendations for RAS in current ERAS® and national guidelines likely reflects several unresolved issues. These include the limited number of randomized controlled trials, the heterogeneity of available robotic systems, and a continuing debate regarding cost-effectiveness and resource allocation in an aging society. Although robotic systems provide superior visualization and technical precision, the initial investment, maintenaince costs and probably extended operative times during the learning curve, remain relevant considerations, particularly when caring for multimorbid patients. Furthermore, future cost benefit analysis is needed to determine whether the potential clinical advantages of RERAS justify these expenditures on a broader scale (38, 39).

Beyond technical and economic aspects, postoperative outcomes in elderly patients are strongly influenced by frailty, sarcopenia and nutritional status. These factors can't be captured by chronological age or ASA classification alone. Frailty has been recognized as an independent predictor of postoperartive complications and delayed recovery (40). Marana demonstrated in 2022 that simple functional measures such as handrgrip strength can serve as reliable markers of frailty and predict length of hospital stay after abdominal surgery (41). Incorporating standardized frailty assessement into enhanced recovery programs may therefore allow for more and individualized prehabilitation and perioperative risk adjustment.

Nutritional and immunonutrional strategies also represent essential elements of an optimized ERAS® approach in older adults. Boccardi reported in 2024 that targeted perioperativ supplementation with protein, amino acids and micronutrients can mitigate surgical stress, preserve muscle mass and enhance postoperative recovery (42). Similarly, Cozza emphasized in 2025 the importance of tailored transfusion and anemia management in elderly patients undergoing surgery in order to reduce complications (43). These principles are consistent with the ERAS® concept of perioperative stress modulation through multimodal interventions and underline the need to expand current protocols to strengthen structured prehabilitation, nutrional optimization and frailty screening.

Our results contribute to this growing evidence by providing real world-data on the feasibility of RERAS implementation in older patients. Nevertheless, further prospective studies, ideally randomized controlled or prospensity matched, are required to determine wheter the physiological and functional benefits of RAS translate into measurable improvements in recovery and quality of life among frail populations undergoing surgery.

Looking ahead, future improvements in colorectal cancer care may not only rely on surgical refinement but also on technological advances in diagnostics. Artificial intelligence (AI), and particularly deep learning, is gaining traction across diagnostic modalities, from endoscopy and imaging to histopathological classification.

Bousis recently highlighted how AI systems can support early and accurate detection of colorectal tumors across the full spectrum of diagnostic tools (44). Meanwhile, Chlorogiannis demonstrated that convolutional neural networks can classify histological slides with near-expert accuracy and extract prognostically relevant information from standard Haematoxylin and Eosin images (45).

While not yet standard in clinical practice, these tools may soon enhance risk stratification and support individualized treatment planning, especially in complex patient cohorts like older adults.

While the outcomes are positive, it is crucial to view our findings with caution. The current learning curve likely contributes to the relatively high reoperation rate and may account also for the relatively high minor non-surgical complication rate following rectal resections. It also remains unclear whether the reoperations are predominantly due to newly trained robotic surgeons or if even experienced robotic surgeons face similar complication rates. The analysis also does not address whether higher comorbidities directly correlate with increased postoperative complications in this patient cohort. Given the absence of a comparison group (e.g., laparoscopic or open resections within an ERAS® protocol, or RAS cases outside an ERAS® pathway), our findings should be interpreted as descriptive observational data from two high-volume centers, rather than as evidence of superiority or inferiority of the approach. This warrants future investigation through additional studies.

## Conclusion

Each hospital operates within its own infrastructure, resources, and capabilities. Consequently, the implementation of new treatment concepts varies across institutions, with each hospital encountering its own successes and challenges. However, in pursuit of optimal patient care, evidence-based measures remain the gold standard. The ERAS® concept has already demonstrated its ability to improve patient outcomes through evidence-based practice. The challenge, therefore, lies in showing that robotic surgery can be introduced alongside such a multimodal treatment approach without the learning curve adversely affecting patient care.

Our analysis suggests that combining ERAS® with robotic colorectal surgery may be implemented safely in elderly patients in this setting. While this combined approach has the potential to enhance patient care through minimally invasive, evidence-based protocols tailored to the complex needs of an aging population, it should be noted that this was solely a descriptive, retrospective two-center cohort focusing on our institutional experience, with no direct comparisons to alternative techniques.

Further prospective, ideally randomized or prospensity-matched studies are required to determine wheter the observed outcomes are attributable to the robotic approach itself, ERAS® compliance or to institutional expertise. Such investigations will be essential to define the precise role of this combined concept, termed RERAS, within future ERAS® frameworks for geriatric surgical care.

## Data Availability

The raw data supporting the conclusions of this article will be made available by the authors, without undue reservation.
